# Pregnancy Outcomes Following Artificial Insemination and Embryo Transfer in Dairy Cows—Expectations vs. Reality

**DOI:** 10.1111/rda.70283

**Published:** 2026-09-02

**Authors:** Ana Clara Canto Souza, Pat Lonergan

**Affiliations:** ^1^ School of Agriculture and Food Science University College Dublin Dublin 4 Ireland

**Keywords:** AI, ET, fertility, IVP, pregnancy loss

## Abstract

Bovine embryo transfer (ET) has been a consistently growing market worldwide with in vitro produced (IVP) embryos now far outnumbering those produced by traditional superovulation. Because transfer of a viable blastocyst‐stage embryo to the uterus bypasses many of the potential biological causes of early pregnancy failure associated with artificial insemination (AI; anovulation, sperm transport, fertilization failure, early embryonic death), pregnancy success should logically be higher following ET than following AI. However, this is not always the case. One of the main factors contributing to this paradox is pregnancy loss following ET, particularly with IVP embryos. This greater embryo mortality presents a significant obstacle to more widespread use of IVP embryos. Improvements in embryo culture media as well as in our understanding of the underlying physiological and molecular regulation of early embryo development and embryo‐maternal communication leading to a successful pregnancy will significantly contribute to improved reproductive efficiency and sustainability in agri‐food production.

## 1 Introduction

Reproductive performance underlies efficient dairy herd productivity, influencing not only replacement rates and milk production but also the economic sustainability of dairy farms (Cabrera 2014; Hanrahan et al. 2018). A successful pregnancy, resulting in the birth of a healthy calf, requires a series of well‐coordinated physiological events from fertilization to term; however, despite many technological advances, reproductive efficiency is still challenged by the occurrence of embryonic and fetal loss. A substantial proportion of reproductive failure occurs before pregnancy can be reliably diagnosed, and even after confirmation, additional losses compromise final calving outcomes (Berg et al. 2022; Domingues et al. 2023; Wiltbank et al. 2016).

**FIGURE 1:**
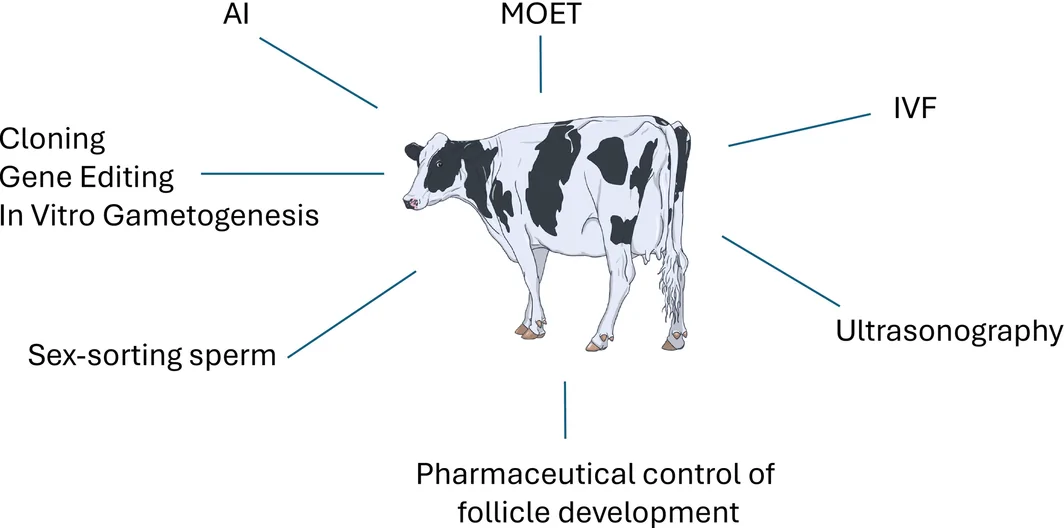
Assisted reproductive technologies available to dairy cattle breeders.

In the toolbox of assisted reproductive technologies (ARTs) available to breeders (Figure 1), both artificial insemination (AI), with conventional and more recently, sex‐sorted semen, and to a lesser extent, embryo transfer (ET) have transformed cattle breeding by accelerating genetic gain, enabling dissemination of superior genetics, and allowing more precise reproductive management (Crowe et al. 2021; Lonergan et al. 2025; Lucy 2025). Artificial insemination remains the most universally used reproductive biotechnology in dairy cattle, enabling widespread dissemination of elite sire genetics. By combining AI with synchronization protocols and sex‐sorted semen, producers can manipulate time of insemination and offspring sex ratio, thereby enhancing genetic progress and the proportion of replacement heifers of high genetic value (Butler et al. 2023).

Embryo transfer offers additional benefits by increasing the reproductive output from superior dams and bypassing the earliest and most vulnerable stages of embryonic development (Baruselli et al. 2020; Hansen 2020b). In doing so, ET has the capacity to intensify genetic gain from superior maternal genetics while reducing calving intervals and enhancing herd performance (Hansen 2020a). Moreover, reproductive strategies that integrate beef‐on‐dairy objectives are gaining traction as a means of adding value to dairy calf crops (Butler et al. 2023; Lonergan et al. 2025). In vitro embryo production (IVP) offers several advantages over traditional multiple ovulation embryo transfer (MOET), including more embryos per unit time, more flexibility in sire selection and the option to use it on prepubertal donors to accelerate generation interval (Table 1).

**TABLE 1:** Hypothetical embryo production and overall yield per donor considering the same 3‐month period, using multiple ovulation and embryo transfer (MOET) or ovum pick‐up with in vitro embryo production (OPU‐IVP) in dairy cows, based on field data (Crowe et al. 2024; Quinton 2025). The adoption of OPU‐IVP amplifies the number of replacement females potentially produced in the same period due to the frequency of sessions and more efficient use of sex‐sorted semen.

Parameters	MOET	OPU‐IVP
Period	3 months	3 months
Frequency of sessions	1 every 45 days	1/week
Total sessions	2	12
Embryos/session	6	4
Total embryos	12	48
Pregnancy/session^a^	4	2
Total pregnancies	8	24
Total calves^b^	7	19
Heifer calves^c^	3	17

^a^ Assumes a pregnancy rate of 60% for MOET and 50% for IVP‐ET on Day 35.

^b^ Assumes a pregnancy loss of approximately 10% for MOET and 20% for IVP‐ET between Day 35 and calving (based on Crowe et al. 2025b).

^c^ Assumes 90% sex bias towards females using X‐sorted semen. Sex‐sorted semen is not typically used in MOET.

The refinement of ARTs has grown in parallel with knowledge of reproductive physiology in cattle, and pregnancies generated by AI and ET have served as important experimental models to expand that knowledge (Crowe et al. 2025b; Sales et al. 2025; Sartori et al. 2023). While AI depends on ovulation of a competent oocyte, successful fertilization, and early embryonic development in the oviduct, ET bypasses issues related to follicle development, oocyte quality, fertilization, and early cleavage by transferring a viable embryo directly to the uterus. One could therefore reasonably assume that pregnancy rates with ET would be superior to those achieved with AI; however, that is not always the case in practice (Hansen 2020a, 2023). This review explores the gap between theoretical promise and commercial reality and summarizes recent data on pregnancy outcomes following AI and transfer of IVP embryos in lactating dairy cows.

## 2 Pregnancy Establishment

**FIGURE 2:**
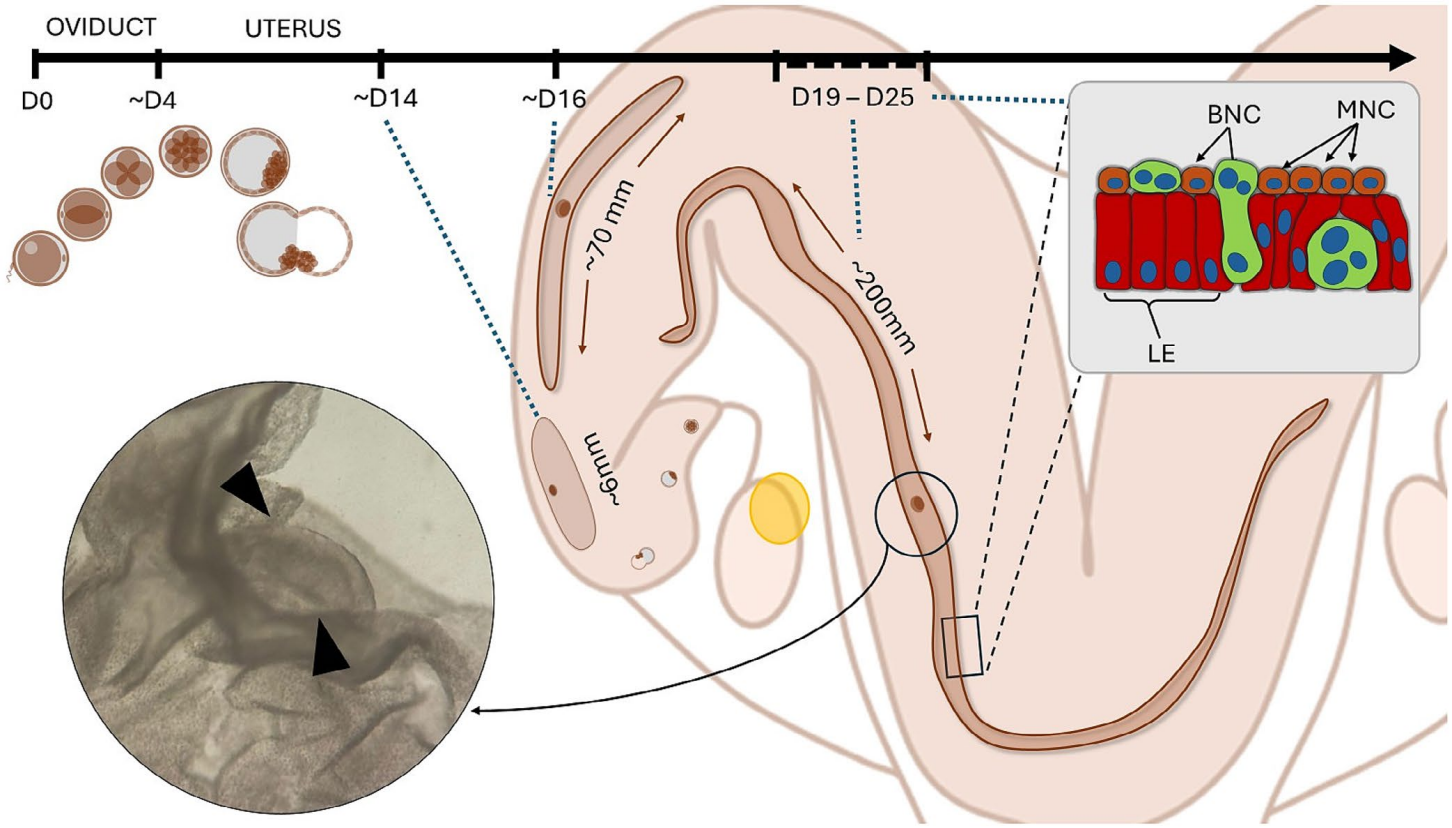
Schematic timeline and key milestones of bovine embryo development from fertilization to conceptus attachment. Early cell divisions occur in the oviduct, and the embryo reaches the uterus around Day 4 (D4) after fertilization. Following hatching from the zona pellucida, the embryo enters a phase of rapid growth characterized by trophectoderm proliferation. During this period, the conceptus changes morphology from an ovoid to tubular and then filamentous structure. Throughout elongation, the increasing number of trophectoderm cells produce interferon‐tau (IFNT), which coincides with the period of maternal recognition of pregnancy. Around this time, a subset of mononucleate trophectoderm cells (MNC) differentiates into binucleate cells (BNC). Conceptus attachment occurs around D20, beginning with apposition to the luminal endometrial epithelium (LE), followed by migration of BNC between LE cells. As pregnancy progresses, BNC fuse with LE cells to form syncytial plaques, a defining feature of the ruminant placenta. Arrowheads indicate D16 embryonic disc observed under a stereomicroscope.

Successful establishment of pregnancy in cattle, as in all mammals, requires complex signalling between both the developing conceptus (embryo and extraembryonic membranes) and the uterine endometrium to ensure normal conceptus growth and development. Following fertilization in the oviduct, the early embryo undergoes the first mitotic cleavage divisions before entering the uterus at around the 16‐cell stage on approximately Day 4 after ovulation. It soon forms a morula and, around Day 7, a blastocyst, containing an inner cell mass and a single layer of trophectoderm surrounding a fluid‐filled blastocoel cavity. In contrast to human and rodent embryos, which implant soon after hatching from the zona pellucida, ruminant conceptuses remain free‐floating for the first 2 to 3 weeks before implantation (Figure 2).

After hatching on approximately Days 8–9, the spherical blastocyst grows and changes in morphology from a spherical to ovoid shape during a transitory phase preceding the characteristic elongation of the trophectoderm to a filamentous form that begins between Days 12 and 14 (Hue et al. 2012). The inner cell mass differentiates to form the epiblast (which gives rise to the embryo proper) and the hypoblast (which gives rise to the yolk sac) while the trophectoderm gives rise to the placenta (Frankenberg et al. 2016). As the conceptus elongates and attaches to the endometrial luminal epithelium (LE), on around Day 20 in cattle (Wathes and Wooding 1980), it synthesizes and secretes type 1 interferon tau (IFNT), the signal for maternal recognition of pregnancy in ruminants (Bazer and Thatcher 2017; Forde and Lonergan 2017). IFNT acts on the endometrial LE and superficial glandular epithelium to inhibit luteolytic pulses of prostaglandin F2α that would result in the structural and functional termination of the ovarian corpus luteum. The result is maintenance of the corpus luteum, the source of progesterone, which is unequivocally required for conceptus development and the maintenance of pregnancy (Lonergan and Sanchez 2020). Recently, Wolf et al. (2026) reported preliminary data describing the successful establishment of pregnancies following transfer of IFNT‐null embryos, produced via genome editing and somatic cell nuclear transfer. This discovery, together with reports of pregnancy in Type I interferon receptor (IFNAR2) knockout sheep (Davies et al. 2026) challenges the long accepted paradigm of IFNT being indispensable for pregnancy recognition in ruminants and encourages researchers to revisit other molecules and mechanisms, independent of IFNT, involved in embryo‐maternal communication and pregnancy recognition (Mathew et al. 2019; Sanchez et al. 2019; Spencer et al. 2013).

Aberrations during the period of conceptus elongation and attachment are strongly implicated in subsequent embryo loss as developmental programs establish the different embryonic and extra‐embryonic lineages that continue to differentiate over time (Mamo et al. 2011; Pfeffer and Pearton 2012; Scatolin et al. 2024; van Leeuwen et al. 2015). Mononucleate cells (MNC) constitute the majority of the trophoblast cells in the elongating conceptus, are responsible for synthesis and secretion of IFNT and are involved in attachment of the conceptus to the uterine LE for implantation. Binucleate trophoblastic giant cells (BNC) begin to differentiate from the MNC between Days 17 and 19 of gestation and comprise 15%–20% of the trophoblast cells during apposition and attachment phases of implantation (Wathes and Wooding 1980; Wooding 2022). Amongst other molecules, these cells secrete pregnancy‐associated glycoproteins (PAG). After apposition and firm attachment to the LE by Days 20–22, BNC fuse with individual LE cells to form trinucleate foetal‐maternal cells and syncytial plates through which these molecules can be released into the uterine vasculature. Since their first isolation from bovine extra‐embryonic membranes (Butler et al. 1982), PAG have become reliable markers for early pregnancy diagnosis in cattle (Sasser et al. 1986). Indeed, concentrations of PAG in serum can be reliably used to assess BNC development and diagnose pregnancy in cows around Day 25 (Sasser et al. 2009) and low concentrations are associated with cows predicted to undergo late embryonic mortality (Domingues et al. 2023; Pohler et al. 2016; Santos et al. 2023). Although causality has not yet been fully demonstrated, serial daily measurements of PAG have been used to estimate the day of presumptive conceptus attachment (Middleton and Pursley 2019; Pursley et al. 2023), and this strategy revealed that later presumptive attachment (as predicted by the initial increase in PAG concentrations) is strongly associated with later pregnancy loss (Middleton and Pursley 2019; Pursley et al. 2023). After attachment, the placenta continues to develop, forming a fully developed chorioallantoic placenta, reaching high circulating PAG concentrations by Day 30 of pregnancy, when the embryo is fully dependent on the placental connection to the maternal blood supply for nutrition, oxygen, and waste exchange.

## 3 Expectations: Biological Potential of AI and ET

Artificial insemination remains the predominant ART in commercial dairy systems, and its widespread adoption since its introduction in the 1950s has driven genetic improvement (Foote 2002; Lonergan 2018), further amplified by the widespread uptake of sex‐sorted semen to breed replacements (Butler et al. 2023). The introduction of synchronization protocols, such as Ovsynch and its derivatives, has enabled fixed‐time insemination without the need for oestrus detection, thereby improving submission rates, reducing labour costs, and contributing to more uniform conception outcomes across herds (Consentini et al. 2025; Fricke and Wiltbank 2022). Additionally, synchronization protocols allow better alignment of endocrine conditions at ovulation, which can improve early conceptus development (Andrade et al. 2025; Cerri et al. 2009). In high‐input dairy systems, synchronization protocols are often more effective than relying on natural oestrus (Fricke and Wiltbank 2022), which can be explained by a better control of progesterone concentrations during the growth of the ovulatory follicle and controlled duration of follicular dominance (Fricke and Wiltbank 2022; Martins et al. 2018). This scenario reduces the risk of premature meiotic resumption and results in improved oocyte quality and increased likelihood of fertile cycles (Fricke and Wiltbank 2022; Minela et al. 2024). Furthermore, by maximizing submission rates (all cows can potentially be bred on the first day of the breeding season), synchronization can accelerate herd pregnancy rate, which is critical in season systems with a short, defined breeding season (Hanrahan et al. 2018). Cows that conceive within the optimal window exhibit shorter calving intervals and lower body condition gain towards the end of lactation. Consequently, these animals experience a lower incidence of postpartum metabolic disorders, improved fertility at first insemination, and reduced pregnancy loss in the subsequent lactation (Fricke et al. 2023; Middleton et al. 2019). Thus, the strategic use of AI and synchronization protocols acts not only as a breeding tool but as a management strategy to mitigate the metabolic challenges inherent to the transition period.

In combination with genomic selection, sex‐sorted semen allows rapid dissemination of superior genetics and more efficient herd expansion (Butler et al. 2023). In addition, reducing the production of male calves in dairy farms addresses the growing concern about their welfare (Bolton et al. 2026; Maher et al. 2021; Rutherford et al. 2021). Although the market value of these animals may vary, dairy‐breed steers have inferior carcass conformation compared to beef‐breed steers (Berry et al. 2018). ‘Beef‐on‐dairy’ is currently a hot‐topic, involving the use sexed semen to inseminate the best dams to generate replacements and use of beef semen or beef embryos on all remaining dams. This strategy results in accelerated genetic gain and the transformation of the dairy herd calf crop to a combination of high genetic merit dairy female calves and premium quality beef calves (Berry 2021; Crowe et al. 2021).

While AI maximizes the dissemination of elite paternal genetics, ET provides a maternal parallel by amplifying the impact of superior dams. The use of ET, in particular, with IVP embryos, allows for the accelerated propagation of genetically elite females by increasing the reproductive output of superior dams and bypassing the earliest and most vulnerable stages of embryonic development (Baruselli et al. 2020). According to the annual reports of the International Embryo Technology Society (IETS), the use of IVP embryos first surpassed that of in vivo–derived (IVD) embryos (produced by superovulation) nearly a decade ago and the gap between the two methods of embryo production has increased steadily since then. For the first time in 2024, the production of bovine embryos in vitro reached a historical milestone, with more than two million embryos produced worldwide (Viana 2025). Notably, approximately 42% of transferred IVP embryos were cryopreserved (frozen–thawed or vitrified–warmed). This highlights the central role of cryopreservation technologies in enabling large‐scale dissemination, international trade, and flexible use of IVP embryos across diverse production systems. Associated with synchronization protocols for recipients, the use of cryopreserved embryos supports reproductive planning in short breeding seasons and in seasonal systems (Crowe et al. 2024).

Hansen (2020b) reviewed the central paradox of ET in terms of its success: because transfer of a viable blastocyst‐stage embryo to the uterus bypasses many of the potential biological causes of early pregnancy failure associated with AI (anovulation, sperm transport, fertilization failure, early embryonic death), pregnancy success should logically be higher following ET than following AI. Indeed, the consequences of well‐described lactation‐induced metabolic stress on the follicle, oocyte, and oviduct function are simply bypassed when ET is used. Nonetheless, pregnancy outcomes following ET are not consistently better than those achieved with AI except in the case of cows under heat stress and repeat‐breeders (Hansen 2020a; Oliveira et al. 2019; Rodrigues et al. 2011). Understanding some of the factors underlying this discrepancy is important so that the gap between expectations and the reality associated with ET is narrowed.

## 4 Reality: Field Outcomes and Pregnancy Loss After AI and ET

Notwithstanding the benefits of AI and ET highlighted above, reproductive outcomes in dairy cows continue to reflect important biological constraints. The transition period in high‐yielding dairy cows involves substantial metabolic adaptations required to sustain milk production, including increased dry matter intake, elevated hepatic blood flow, altered insulin and IGF‐1 dynamics, and enhanced steroid hormone clearance (Sangsritavong et al. 2002; Sartori et al. 2010). These changes substantially modify the endocrine milieu in which ovulation, fertilization, and early embryonic development occur. We have previously shown that the reproductive tract of the lactating dairy cow is compromised in its ability to support early embryo development compared with that of maiden heifers (Rizos et al. 2010) or age‐matched nonlactating cows and this may contribute to early embryo mortality observed in such animals (Maillo et al. 2012). Increased hepatic metabolism of progesterone in high‐producing cows reduces circulating luteal concentrations compared with heifers or dry cows, particularly during the early luteal phase (Sangsritavong et al. 2002). Adequate progesterone during the first 5–7 days after ovulation is critical for uterine histotroph production, endometrial gene expression, and conceptus elongation (Forde et al. 2014; Forde and Lonergan 2017; Sanchez et al. 2018). Consequently, suboptimal luteal function can compromise the uterine environment required for embryo survival (Forde et al. 2014; Forde and Lonergan 2017; Wiltbank et al. 2016). Consistent with these findings, cows in the lowest progesterone quartile at Day 7 exhibit reduced P/AI and P/ET along with a higher risk of pregnancy loss before Day 18 compared to those with higher concentrations (Crowe et al. 2025a).

**FIGURE 3:**
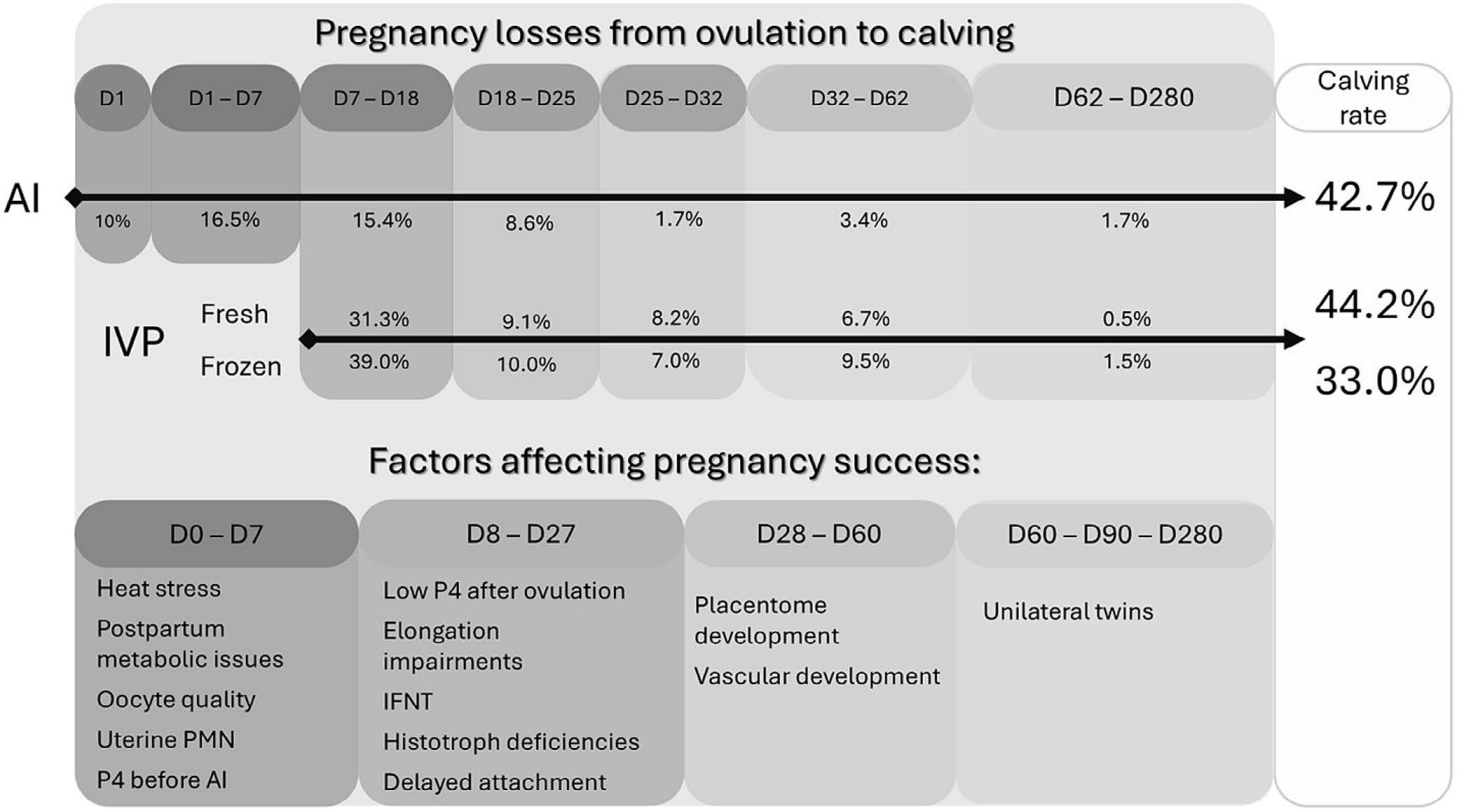
Key periods of pregnancy loss in cattle and the incidence of loss according to embryo origin, from ovulation (D0) until term (D280). Wiltbank et al. (2016) defined critical periods of pregnancy loss associated with specific factors. Crowe et al. (2025b) evaluated pregnancy loss using different diagnostic approaches in pregnancies generated by artificial insemination (AI) or by transfer of in vitro–produced (IVP) embryos, either fresh or frozen–thawed. Most pregnancy loss occurs during the early stages of development, particularly before day 28, when embryonic failure can be linked back to environmental conditions, maternal health, or embryonic development competence. Incidence of pregnancy loss reduces drastically after the second trimester. Identifying when pregnancy loss occurs is essential for linking failure to specific physiological events and developmental milestones. PMN: Polymorphonuclear cells; P4: Progesterone; IFNT: Interferon‐tau.

Representative studies on pregnancy loss after AI and ET are summarized in Table 2, whereas Figure 3 details the incidence and the mechanisms driving these losses at different timepoints. The largest proportion of pregnancy loss occurs within the first week following AI (Berg et al. 2022; Crowe et al. 2025a; Wiltbank et al. 2016). Berg et al. (2022) used a combination of morphological examination of embryos on Days 7 and 15 and pregnancy diagnosis by ultrasonography on Days 28, 35, and 70 and reported P/AI success rates of 70.9%, 59.1%, 63.8%, 62.3%, and 56.7% at Days 7, 15, 28, 35, and 70, respectively, in seasonal‐calving dairy cows managed under a pasture‐based system. Fertilization failure (15.8%) and embryonic arrest before the morula stage (10.3%) were identified as the major developmental events that contributed to pregnancy losses during the first week after oestrus, and conceptus elongation failure (7%) contributed to pregnancy failure during the second week after oestrus. Considering that ET involves transferring a viable embryo to a recipient at a stage of the cycle that is synchronous to the embryo, circumventing oocyte and fertilization issues that could impact the outcome of AI, it is reasonable to expect that pregnancy rates would be higher than AI, but that is not always the case (Hansen 2020b).

**TABLE 2:** Comparison of pregnancy per service event (P/S) and pregnancy loss (PL) in dairy cows using artificial insemination (AI) or embryo transfer of in vitro‐produced (IVP) or in vivo‐derived (IVD) bovine embryos. Period refers to the day (D) of first and last pregnancy diagnosis used to calculate the proportion of losses. Superscript letters indicate a statistical difference between groups.

References	Service event (*n*)	P/S	PL	Period
Stewart et al. (2011)	AI (227)	18.3%	11.0%	D40 – D97
	IVP fresh (216)	29.3%	25.9%	
	IVP vitrified (279)	45.5%	20.4%	
Viziack et al. (2017)	AI (21706)	—	23.5%	D60 – Birth
	IVP (9588)	—	26.5%	
	IVD (3754)	—	26.9%	
Edelhoff et al. (2020)	IVP 1st ET (674)	42.3%	26.3%	D31 – D59
	IVP 2nd ET (432)	44.9%	18.5%	
Crowe et al. (2024)	AI (243)	48.8%	4.7%	D32 – D62
	IVP fresh (435)	56.1%	13.3%	
	IVP frozen (426)	42.9%	20.2%	
Vandresen et al. (2024)	AI heifers (18681)	38.0%	18.4%	D28 – D40
	IVP heifers (5341)	41.4%	35.7%	
	AI cows (42036)	36.4%	34.2%	
	IVP cows (9120)	33.9%	47.7%	
Crowe et al. (2025b)	AI conventional semen (136)	51.5%	16.4%	D19 – Birth
	AI sex‐sorted semen (136)	56.6%	30.7%	
	IVP frozen (136)	55.9%	33.8%	
Lamim et al. (2025)	IVP (274)	35.4%	24.7%	D32 – D60
Murphy et al. (2025)	IVP sex‐sorted semen (250)	57.9%	9.9%	D32 – D63
	IVP Conventional semen (218)	58.8%	14.1%	

We recently carried out a large controlled field trial to compare pregnancy success and pregnancy losses in lactating Holstein‐Friesian cows that received AI or ET using fresh or frozen IVP embryos from either dairy or beef breeds (Crowe et al. 2024). A total of 1106 cows were enrolled, with 863 receiving ET and 243 undergoing AI. Oocytes were collected from elite dairy and beef donors weekly by ovum pick‐up, as well as from commercial beef heifers post‐slaughter. Pregnancy rates on Day 32 were similar between AI (48.8%) and ET (48.9%) and between dairy and beef embryos (50.3% vs. 48.1%, respectively), but significantly less for cows receiving frozen embryos (41.6%) compared with fresh embryos (56.1%). Pregnancy loss between Days 32 and 62 was significantly greater for ET (15.1%) than AI (4.7%).

We subsequently characterized the incidence and timing of pregnancy loss from service event (AI or ET) to calving in the same cohort of lactating Holstein‐Friesian cows (Crowe et al. 2025a). Consistent with previous publications (Berg et al. 2022; Sartori et al. 2010; Wiltbank et al. 2016), most embryonic loss occurred early after fertilization, irrespective of whether AI or ET was used. The predicted probability of pregnancy (%) varied at each time point (Day 7, 18, 25, 32, 62, 125, parturition) depending on treatment (AI: 77.0, 60.2, 52.3, 48.8, 47.0, 44.6, 44.0; fresh ET: 100.0, 69.5, 60.3, 56.1, 48.4, 46.8, 45.5; frozen ET: 100.0, 61.7, 52.2, 41.6, 32.9, 31.8, 30.2). Irrespective of treatment, the largest proportion of pregnancy loss occurred in the period from service event (AI on Day 0 or ET on Day 7) to Day 18. There was a greater probability of pregnancy loss between Day 32 and 62 following ET (fresh: 11.3%, frozen: 18.0%) than AI (4.0%), with minimal loss occurring between Day 62 and parturition (AI: 1.8%, fresh ET: 1.9%, frozen ET: 3.5%). Treatment differences in the predicted probability of pregnancy were detected between fresh ET versus frozen ET on Day 32 and both AI and fresh ET versus frozen ET on Day 62, 125, and at parturition. The percentage of cows that calved following transfer of a fresh embryo (45.5%) was similar to AI (44.0%), but less when a frozen embryo was transferred (30.2%), illustrating the challenges that have yet to be surmounted if ET is to achieve its full potential.

The underlying mechanisms responsible for such loss are not clear but are likely related to the consequences of suboptimal post‐fertilization culture conditions on blastocyst quality (Lonergan, Rizos, Gutierrez‐Adan, et al. 2003; Lonergan, Rizos, Kanka, et al. 2003; Rizos et al. 2002). Although media composition for the in vitro production of embryos is constantly evolving, the suboptimal environmental conditions inherent to in vitro culture lead to suboptimal embryo quality, particularly after cryopreservation (Crowe et al. 2025b; Sanches et al. 2016; Sanches et al. 2017). IVP embryos differ in terms of morphology, ultrastructure, cryotolerance, and transcriptome from those derived in vivo (Lonergan and Fair 2008), leading to a compromised ability of the resulting conceptus to appropriately signal to the maternal endometrium during elongation and attachment (Bauersachs et al. 2009; Mansouri‐Attia et al. 2009; Sanchez et al. 2019). Appropriate molecular interaction between conceptus and endometrium is an important feature in the period leading up to attachment (Biase et al. 2023; Moraes et al. 2018; Sanchez et al. 2019), and dysregulation of conceptus‐maternal communication is a probable cause of lower calving rates after the transfer of IVP embryos. For example, delayed conceptus elongation results in short conceptuses which secrete much less IFNT (Rizos et al. 2012) and fail to elicit an appropriate transcriptional response from the endometrium compared to long conceptuses (Sanchez et al. 2019). Furthermore, evidence of delayed embryonic attachment has been related to reduced fertility and pregnancy losses in subsequent phases of development (Middleton and Pursley 2019; Pursley et al. 2023). Recently, using daily measurement of PAGs to predict presumptive conceptus attachment, Crowe et al. (2025a) identified that the transfer of frozen–thawed IVP embryos resulted in later attachment compared to conventional AI. This evidence of delayed attachment could explain, at least partially, the increased risk of pregnancy loss in IVP embryos. Further research is required to fully elucidate how the interaction between embryo quality, cryopreservation and uterine receptivity can be optimized to improve the efficiency of IVP technologies.

## 5 Future Perspectives

Bovine ET has been a consistently growing market worldwide with IVP embryos now far outnumbering those produced by traditional superovulation (Viana 2025); however, pregnancy loss after ET is still a major barrier to achieving the full potential for this technique. Increasing the yield of transferrable embryos through improvements in embryo culture conditions to increase the competence of the embryo to establish pregnancy (and minimize pregnancy loss) is crucial. Other constraints to the more widespread adoption of IVP technology include the cost, which may exceed gains due to genetic improvement or perceived improvements in fertility, availability of oocytes from elite females, and number of transferable embryos produced per OPU session. The latter may be overcome through improved donor selection, perhaps in combination with assessment of ovarian reserve through Anti‐Müllerian Hormone measurement or antral follicle counts. Future developments in stem cell biology may make it possible to generate gametes from stem cells as has been reported for mice (Saitou and Hayashi 2021) and allow so‐called in vitro breeding, where future generations are turned over in a petri dish until a desirable point in terms of genotype is reached (Goszczynski et al. 2019; Goszczynski et al. 2023).

While we already know quite a lot about embryo maternal communication, improved understanding of the molecular regulation of embryo development and the reciprocal communication between the maternal uterus and the developing conceptus during pregnancy establishment will contribute to reducing pregnancy loss. Post hatching culture systems that support normal development of the early stages of elongation are allowing us to better understand the regulation of conceptus elongation and gastrulation (Martinez de Los Reyes et al. 2026). Lastly, application of machine learning to the myriad of publicly available datasets describing the transcriptome of embryos produced under varying conditions to identify genes predictive of embryonic competence (Rabaglino et al. 2023; Rabaglino and Hansen 2024) or endometrial biomarkers of pregnancy success (Hoorn et al. 2024) may lead to improvements in pregnancy maintenance.

## Author Contributions

**Ana Clara Canto Souza:** conceptualization, formal analysis, investigation, writing – original draft, writing – review and editing, visualization, supervision, project administration, funding acquisition; **Pat Lonergan:** conceptualization, formal analysis, investigation, writing – review and editing, visualization, supervision, project administration, funding acquisition.

## Funding

This work was supported by: HORIZON EUROPE Marie Sklodowska‐Curie Actions (10.13039/100018694)*101169308 Department of Agriculture, Food and the Marine, Ireland (10.13039/501100001584)*2021R665.

## Conflicts of Interest

The authors declare no conflicts of interest.

## Data Availability

The data that were used to generate review come from published papers in the literature and can be accessed through those papers listed in the references.
